# SARS, the First Pandemic of the 21st Century^1^

**DOI:** 10.3201/eid1011.040797_02

**Published:** 2004-11

**Authors:** James W. LeDuc, M. Anita Barry

**Affiliations:** * Centers for Disease Control and Prevention, Atlanta, Georgia, USA;; † Boston Public Health Commission, Boston, Massachusetts, USA

**Keywords:** SARS, 21^st^ Century Pandemic

The 2003 outbreak of severe acute respiratory syndrome (SARS) shocked the world as it spread swiftly from continent to continent, resulting in >8,000 infections, with approximately 10% mortality, and a devastating effect on local and regional economies. Three laboratories—one each in Hong Kong, Germany, and the Centers for Disease Control and Prevention (CDC) in Atlanta, Georgia, USA—nearly simultaneously isolated an apparently new coronavirus as the cause of SARS. Through traditional virus isolation and molecular techniques, CDC's team recovered the virus from specimens and characterized it as a novel coronavirus. Specific nucleotide sequences of the new virus were identified in specimens from SARS patients, and an immune response to the agent was demonstrated in patients' sera.

The potential for global spread of SARS was quickly recognized by the World Health Organization (WHO). The Global Outbreak Alert and Response Network was activated to help identify and deploy volunteers from around the world to assist the most severely affected nations, and WHO rapidly issued several recommendations to help nations control outbreaks and prevent spread.

Hong Kong was among the first cities affected by SARS, and its healthcare community suffered greatly from the disease. Some lessons from their experiences included recognition of the value of real-time information in a rapidly progressing epidemic with a large number of cases and the need for frequent patient updates, challenges of national efforts to maintain entry and exit health screening among international travelers, and implementation of home quarantine as an effective tool to interrupt SARS transmission.

In Toronto, Ontario, Canada, the public health department had responsibility for SARS surveillance and case reporting, investigation and management of possible cases, identification and quarantine of contacts, health risk assessment, and communications, and they were a liaison with hospitals regarding infection control. These were massive responsibilities. Serious practical and legal challenges were encountered as the department successfully implemented quarantine measures for the first time in more than half a century. Daunting challenges were also overcome in disease surveillance and reporting; meeting the needs for accurate, timely information and guidance; and implementing effective infection control practices in healthcare facilities. One of the most important lessons was an awareness of the psychosocial problems among healthcare workers directly involved in facing SARS.

